# Neighborhood Inequities in Tobacco Retailer Density and the Presence of Tobacco-Selling Pharmacies and Tobacco Shops

**DOI:** 10.1177/10901981211008390

**Published:** 2021-04-19

**Authors:** Amanda Y. Kong, Paul L. Delamater, Nisha C. Gottfredson, Kurt M. Ribisl, Chris D. Baggett, Shelley D. Golden

**Affiliations:** 1 University of North Carolina, Chapel Hill, NC, USA

**Keywords:** geography, health inequalities, neighborhoods, policy, tobacco control, tobacco retailer density

## Abstract

Studies document inequitable tobacco retailer density by neighborhood sociodemographics, but these findings may not be robust to different density measures. Policies to reduce density may be less equitable depending on how the presence of store types differs by neighborhood characteristics. We built a 2018 list of probable tobacco retailers in the United States and calculated four measures of density for all census tracts (*N* = 71,495), including total count, and number of retailers per 1,000 people, square mile, and kilometers of roadway. We fit multivariable regression models testing associations between each density measure and tract-level sociodemographics. We fit logistic regression models testing associations between sociodemographics and the presence of a tobacco-selling pharmacy or tobacco shop. Across all measures, tracts with a greater percentage of residents living below 150% of the federal poverty level (FPL) had higher density. A higher percentage of Black residents, Hispanic or Latino residents, and vacant housing was inconsistently associated with density across measures. Neighborhoods with a greater percentage of Black residents had a lower odds of having a pharmacy (adjusted odds ratio [a*OR*] = 0.96, 95% confidence interval [CI; 0.95, 0.97]) and tobacco shop (a*OR* = 0.87, CI [0.86, 0.89]), while those with a greater percentage of residents living below 150% FPL had greater odds of having a tobacco shop (a*OR* = 1.18, CI [1.16, 1.20]). Researchers and policymakers should consider how various measures of retailer density may capture different aspects of the environment. Furthermore, there may be an inequitable impact of retailer-specific policies on tobacco availability.

Tobacco retailer availability is a geographic construct, representing the supply of tobacco retailers in a geographic area, and is often quantified through the use of tobacco retailer density measures (Kong et al., 2020; Valiente et al., 2020). In places with higher tobacco retailer density, individuals have greater smoking intentions, are more likely to start or continue smoking, smoke more cigarettes, and are less likely to quit smoking (Finan et al., 2019; Marsh et al., 2020; Valiente et al., 2020).

Several U.S.-based (Hyland et al., 2003; Peterson et al., 2005; Schneider et al., 2005; Siahpush et al., 2010) and international (Chaiton et al., 2013; Kite et al., 2014; Shortt et al., 2015) studies have documented higher tobacco retailer density in neighborhoods with a greater proportion of some shared demographic and social characteristics, potentially putting residents of these neighborhoods at a higher risk of smoking. However, few U.S. studies have assessed neighborhood inequities in tobacco retailer density at the national level. Using census tract sociodemographic characteristics from nearly all census tracts in 2000, Rodriguez et al. (2013, 2014) found that the proportion of Hispanic residents, Black residents, families living in poverty, and urbanicity were each uniquely and positively associated with the number of tobacco retailers per 1,000 people. However, no study of this scope has been completed with more recent data.

The first purpose of this study is to assess and compare associations of four common measures of tobacco retailer density with recent (2018) tract-level sociodemographic characteristics in the United States. There is substantial variation in how researchers and policymakers measure retailer density (Valiente et al., 2020) with little discussion on why specific measures were chosen. Some common density measures include the number of tobacco retailers per population, per land area, or per kilometers of roadway. There has also been little comparison across density measures both within and between studies (Mayers et al., 2012). As a result, conclusions about area-level sociodemographic inequities may not be robust to different measures, potentially under- or overestimating inequities. Additionally, understanding whether findings are similar across measures may be useful for past and future study comparison purposes.

Studies in select U.S. cities and states also suggest that the impact of policies that prohibit pharmacies from selling tobacco products may not be equitable (Craigmile et al., 2020; Giovenco et al., 2019; Hall et al., 2019; Kulbicki & Leslie, 2015). Additionally, some places have implemented policies that only allow specialty tobacco shops to sell tobacco products or certain types of tobacco products (CounterTobacco.org, 2020). While a study of U.S. vape shop density documented some area-level racial, ethnic, and socioeconomic inequities (Dai et al., 2017), it is not known if there are inequities in the availability of all specialty tobacco shops (e.g., vape shops *and* smoke shops), which are common tobacco control policy targets. The second purpose of this study is to assess whether the presence of two tobacco retailer types (i.e., pharmacies, tobacco shops) that are commonly targeted in policies to reduce tobacco retailer density is associated with neighborhood sociodemographic characteristics.

## Method

### Neighborhood Sociodemographic Characteristics

We conceptualized race and ethnicity as a social construct, resulting from racialization and discriminatory systems that create and sustain group-based hierarchies that advantage and disadvantage certain groups (Inwood & Yarbrough, 2010; Krieger et al., 2015; Pratto et al., 2006). Both individuals and places are racialized, resulting in spatially patterned racial inequities in the distribution of resources, health behaviors, and health outcomes (Inwood & Yarbrough, 2010; Lipsitz, 2007; Neely & Samura, 2011). To measure this construct, we used U.S. Census Bureau 2014–2018 American Community Survey population estimates, which measure the census tract-level percentage of the population in different racial and ethnic groups (non-Hispanic Black or African American [Black], Hispanic or Latino), based on self-report survey categories.

While most studies document inequities in retailer density by median household income or federal poverty level (FPL), very few studies have expanded beyond these economic measures. Vacant housing may be an indicator for residential or neighborhood stability: A lack of residential stability may reflect low attachment to a community, potentially leading to fewer opportunities for social cohesion and collective efficacy among its residents to empower a healthy community (Aiyer et al., 2015; Browning & Cagney, 2003; Kawachi & Berkman, 2000; Stockdale et al., 2007), such as organizing to prohibit tobacco retailers. In this study, we included two neighborhood economic variables: percentage of the tract-level population that was living below 150% of the FPL and vacant housing units.

We incorporated a measure of tract-level urbanicity as a control variable, using U.S. Department of Agriculture (2016) Rural-Urban Commuting Area Codes Documentation. Each census tract was categorized as urban, large rural city/town, or small and isolated rural town.

### Tobacco Retailer Density

There is no national tobacco retailer licensing system in the United States, so we created a 2018 list of probable retailers that sold tobacco products intended for off-premise consumption, similar to previous studies (Kong et al., 2021; Ribisl et al., 2017; Rodriguez et al., 2013). In short, we used tobacco product sales data from the latest 2017 Economic U.S. Census to identify 11 North American Industry Classification System (NAICS) store type codes (e.g., Convenience Stores, Tobacco Stores, Pharmacies and Drug Stores) that account for approximately 99% of all retail tobacco product sales. Every NAICS code is associated with a subset of Standard Industrial Classification (SIC) codes, which describes the primary business activity of a retailer in more detail. For example, NAICS 453991 (Tobacco Stores) is associated with several SIC codes, including 599302 (Smoke Shops and Supplies) and 599306 (Electronic Cigarettes). Using these NAICS codes and ReferenceUSA, a database of business establishments that contains NAICS and associated SIC codes, retailer name, and geographic indicators for each retailer, we created a 2018 list of probable brick-and-mortar tobacco retailers. Specific retailer subtypes identified through SIC codes that seemed unlikely to sell tobacco products (e.g., craft galleries and dealers, marine services stations) were excluded from the sample. We further refined the list through a text search of store names to exclude those retailers known to not sell tobacco products (e.g., Target, Whole Foods, CVS Pharmacy).

Consistent with common measures in the literature, we operationalized tobacco retailer density in four ways: (1) total count of tobacco retailers, and tobacco retailers per (2) 1,000 people; (3) land area (square mile); and (4) 10 km of roadway. To calculate these measures, we used a spatial join in ArcMap 10.5 to assign each retailer to its respective census tract and then summed the total number of tobacco retailers within each tract. Publicly available roadway data were downloaded from the U.S. Census Bureau.

We calculated both the total number of tobacco-selling pharmacies and the number of tobacco shops in a tract. We then created two binary variables: One variable indicated whether a tract had at least one tobacco-selling pharmacy (=1), while the other variable indicated whether a tract had at least one tobacco shop (=1). As smaller, independent, and compounding pharmacies may be less likely to sell tobacco products (CounterTobacco.org, 2019), we included only those pharmacies that had at least 10 locations nationally. Similar to other work (Lee et al., 2018), we confirmed that the most frequently occurring pharmacy chain brands sold tobacco products (e.g., Walgreens, Duane Reade, Rite Aid, Thrifty White) and excluded those chain pharmacies known not to sell tobacco products (e.g., CVS, Medicine Shoppe). Using data collated by the American Nonsmoker’s Rights Foundation U.S. Tobacco Control Laws Database©, we identified places with policies that prohibit pharmacies from selling tobacco products and then excluded pharmacies that were located in those places. Finally, as current bans on tobacco sales in pharmacies also include stores (e.g., grocery, warehouse) that have a pharmacy counter (Craigmile et al., 2020), we included retailers that were identified as such (e.g., Kroger Pharmacy) and that met the above criteria.

### Analytic Sample

In 2018, there were 72,377 census tracts with a population of at least one person and that had Rural-Urban Commuting Area and roadway data for all 50 states and the District of Columbia. As tracts are intended to range from 1,200 to 8,000 people and our per capita retailer density measure is per 1,000 people, we excluded those tracts with fewer than 1,000 people (*n* = 748). We additionally assessed the distribution of calculated values of density and omitted two extreme outliers (e.g., 410 retailers per square mile) and those tracts with missing sociodemographic data due to Census Bureau suppression (*n* = 132). This resulted in a final analytic sample of 71,495 tracts (98.8% of all populated tracts).

### Analysis

To investigate associations between the four measures of tobacco retailer density and neighborhood sociodemographic characteristics, we fit unadjusted and adjusted multivariable linear regression models that controlled for the other tract-level variables described previously and area urbanicity. Because the majority of census tracts did not have a tobacco-selling pharmacy (72.3%) or tobacco shop (81.2%), and there was little variability in the count distribution for those that did, we fit unadjusted and adjusted logistic regression models to assess relationships between sociodemographic characteristics and two outcome variables: the presence of at least one (vs. none) tobacco-selling pharmacy and the presence of at least one (vs. none) tobacco shop.

All adjusted models also included a state fixed effect to account for potential state-level differences (e.g., historically tobacco growing state, tobacco retailer licensing laws). We did not find evidence of collinearity in multivariable models (average variance inflation factor was 1.34). To aid in interpretability, each sociodemographic variable was scaled to tens (e.g., 13% = 1.3) so that a 1-unit difference in a sociodemographic variable represents a 10–percentage point difference.

## Results

Characteristics of the analytic sample are shown in Table 1. In 2018, there were an estimated 325,884 tobacco retailers. The average number of tobacco retailers in a tract was 4.6. Average retailer density per 1,000 people was 1.11; per square mile was 4.85; and per 10 km of roadway was 1.20. About 28% and 19% of tracts had at least one tobacco-selling pharmacy or tobacco shop, respectively. Comparing density measures, Pearson correlation coefficients were high for retailers per roadway and square mile (*r* = .96), and for total count of retailers and retailers per 1,000 people (*r* = .77). Correlation was low to moderate for all other retailer density combinations (range: .16–.31).

**Table 1.** Sociodemographic and Tobacco Retailer Availability Characteristics of Census Tract Neighborhoods, United States, 2018 (*N* = 71,495).

Characteristics	*M* (*SD*) or %	Range
Demographic characteristics		
% Non-Hispanic Black	13.3 (21.4)	0–100
% Hispanic or Latino	16.5 (21.4)	0–100
% Living below 150% FPL	24.4 (15.7)	0–100
% Vacant housing units	11.7 (10.3)	0–91.4
Urbanicity		
Urban	82.9%	—
Large rural city/town	8.7%	—
Small and isolated rural town	8.5%	—
Tobacco retailer density		
Total count of retailers	4.6 (4.1)	0–55
Retailers per 1,000 people	1.11 (1.07)	0–17.5
Retailers per square mile	4.85 (12.2)	0–281.6
Retailers per 10 km of roadway	1.20 (2.1)	0–46.9
At least one tobacco-selling pharmacy present	27.7%	—
At least one tobacco shop present	18.9%	—

*Note.* FPL = federal poverty level; km = kilometers.

### Tobacco Retailer Density

For all measures of retailer density, unadjusted analyses indicated positive and statistically significant associations for tract-level composition of percentage of Black residents and residents living below 150% FPL (Table 2). Percentage of Hispanic or Latino composition was positive and significant for all density measures except per 1,000 people (Β = −0.01, *p* < .001). For percentage of vacant housing units, associations were positive and significant for total count of retailers and retailers per 1,000 people but negative for retailers per square mile and 10 km of roadway.

**Table 2.** Unadjusted Analyses Testing Census Tract-Level Associations of Percentage Sociodemographics With Measures of Tobacco Retailer Density, United States, 2018 (*N* = 71,495).

Sociodemographic variable	Total count of retailers, B (*SE*)	Retailers per 1,000 people, B (*SE*)	Retailers per square mile, B (*SE*)	Retailers per 10 km of roadway, B (*SE*)
Non-Hispanic Black	0.03 (0.01)***	0.04 (0.00)***	0.51 (0.02)***	0.10 (0.00)***
Hispanic or Latino	0.06 (0.01)***	−0.01 (0.00)***	1.28 (0.02)***	0.25 (0.00)***
Living below 150% FPL	0.40 (0.01)***	0.16 (0.00)***	1.41 (0.03)***	0.27 (0.00)***
Vacant housing units	0.23 (0.01)***	0.24 (0.00)***	−0.51 (0.04)***	−0.14 (0.01)***

*Note.* Tract-level sociodemographic variables were scaled to 10s (e.g., 10% is coded 1.0) so that estimates may be interpreted as the expected difference in tobacco retailer density for a census tract that has a 10–percentage point greater value in the sociodemographic variable. FPL = federal poverty level; km = kilometers.

* *p* < .05. **p < .01. ***p < .001.

In multivariable adjusted models (Table 3), there were positive and significant associations between the percentage of Black residents and land area and roadway retailer density.

**Table 3.** Adjusted Analyses Testing Census Tract-Level Associations of Percentage Sociodemographics With Measures of Tobacco Retailer Density, United States, 2018 (*N* = 71,495).

Sociodemographic variable	Total count of retailers, B (*SE*)	Retailers per 1,000 people, B (*SE*)	Retailers per square mile, B (*SE*)	Retailers per 10 km of roadway, B (*SE*)
Non-Hispanic Black	−0.13 (0.01)***	−0.02 (0.00)***	0.18 (0.02)***	0.04 (0.00)***
Hispanic or Latino	0.02 (0.01)	−0.02 (0.00)***	0.97 (0.03)***	0.17 (0.00)***
Living below 150% FPL	0.41 (0.01)***	0.15 (0.00)***	1.23 (0.04)***	0.24 (0.01)***
Vacant housing units	−0.19 (0.02)***	0.12 (0.00)***	0.00 (0.05)	−0.05 (0.01)***
Urbanicity				
Urban	Reference	Reference	Reference	Reference
Large rural city/town	1.13 (0.06)***	0.19 (0.01)***	−2.95 (0.16)***	−0.54 (0.03)***
Small and isolated rural town	1.75 (0.06)***	0.44 (0.02)***	−3.68 (0.17)***	−0.73 (0.03)***

*Note.* All models controlled for tract-level urbanicity and other sociodemographics (% Black, Hispanic or Latino, living below 150% FPL, vacant housing) and included a state fixed effect. Tract-level sociodemographic variables were scaled to tens (e.g., 10% is coded 1.0) so that estimates may be interpreted as the expected difference in tobacco retailer density for a census tract that has a 10–percentage point greater value in the sociodemographic variable. FPL = federal poverty level; km = kilometers.

* *p* < .05. **p < .01. ***p < .001.

When comparing two census tracts, one of which had a Black composition that was 10 percentage points higher than the other, we would expect to have 0.18 and 0.04 (*p* < .001) more retailers per square mile and 10 km of roadway, respectively, in the tract with the higher percentage of Black residents. However, negative associations were observed between the percentage of Black residents and both total count (Β = −0.13, *p* < .001) and per 1,000 people (Β = −0.02, *p* < .001) measures.

**Figure 1.**
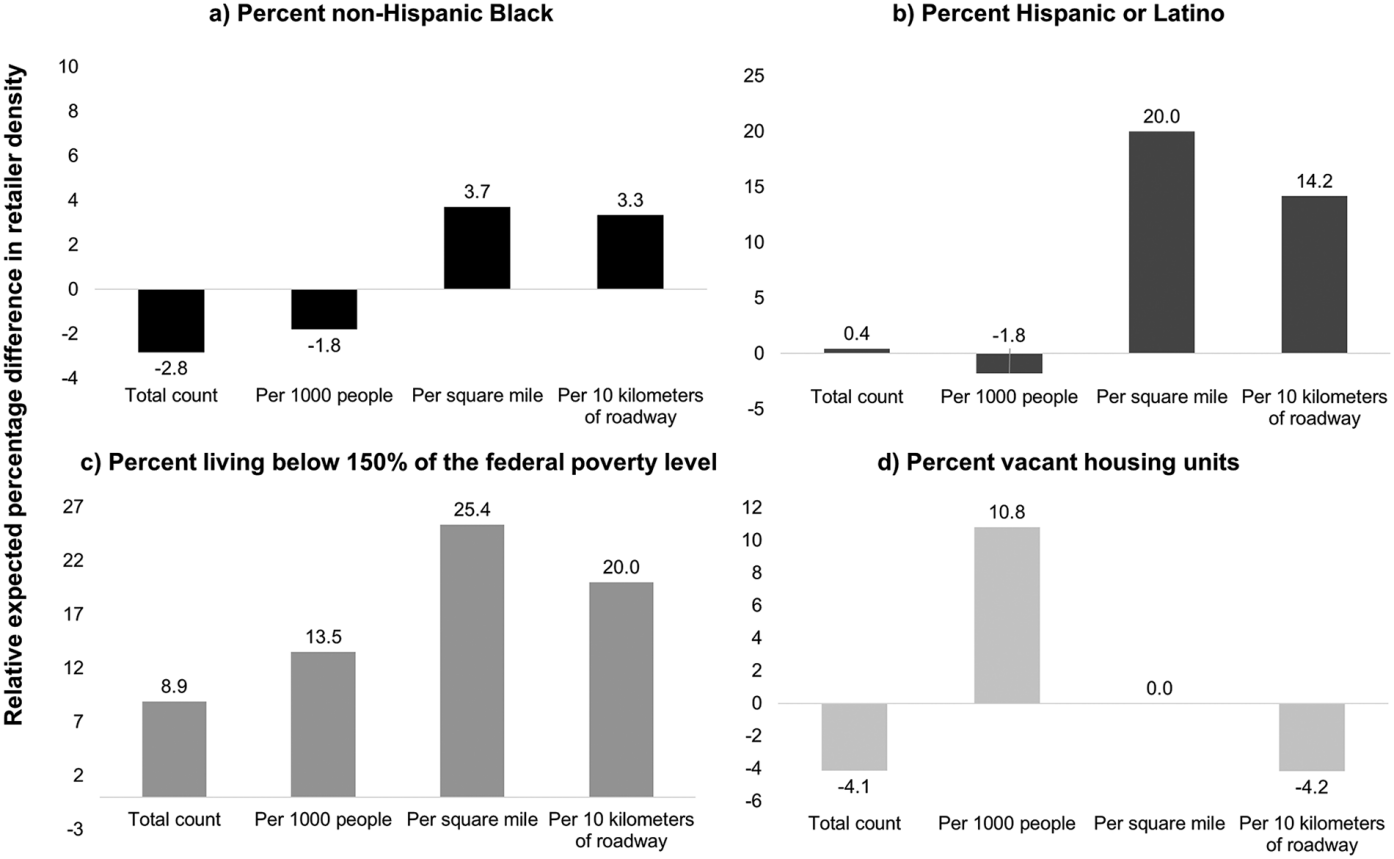
Expected percentage difference in tobacco retailer density relative to average density by tract-level sociodemographic characteristics, United States, 2018 (*N* = 71,495). *Note.* Relative expected percentage differences in retailer density were calculated by dividing the model adjusted parameter estimates (Table 3) by the average retailer density in the sample and then multiplying this number by 100. Tract-level sociodemographic variables were scaled to 10s (e.g., 10% is coded 1.0) so that values may be interpreted as the expected percentage difference in tobacco retailer density (relative to average in sample) for a census tract that has a 10–percentage point greater value in the sociodemographic variable. Only one of the four sociodemographic characteristics (i.e., federal poverty level) shows a consistent pattern across the four density measures. km = kilometers.

Associations for percentage of Hispanic or Latino residents were positive for all retailer density measures except per 1,000 people, which had a negative association (Β = −0.02, *p* < .001). Tracts with a greater percentage of residents living below 150% FPL were associated with higher retailer density across all four measures. Vacant housing was positively associated with retailers per 1,000 people (Β = 0.12, *p* < .001) but inversely associated with total count (Β = −0.19, *p* < .001) and roadway (Β = −0.05, *p* < .001) measures. To help visualize the magnitude of adjusted analyses across retailer density measures, for each sociodemographic variable, we calculated the expected percentage difference in retailer density relative to the average retailer density of the sample (Figure 1).

In a post hoc multivariable analysis, we included interaction terms between each sociodemographic variable and the three-level urbanicity variable. We report simple slope estimates for each urbanicity category and further tested whether associations significantly differed (*p* < .05) between levels of urbanicity (Supplemental Appendix A). In short, we found that the direction and statistical significance of adjusted associations also differed across retailer density measures and urbanicity categories. For example, in tracts designated as small and isolated rural town, the percentage of Black residents was positively associated with the total count of retailers (Β = 0.19, *p* < .05) but was negatively associated in urban tracts (Β = −0.15, *p* < .05). Additionally, among urban tracts and for percentage of Black residents, we documented negative associations for total count and per 1,000 people but positive associations for land area and roadway measures.

### Pharmacies and Tobacco Shop Presence

Finally, we investigated associations between neighborhood sociodemographics and the presence of a pharmacy and the presence of a tobacco shop in a tract (Table 4). Adjusted results indicated that neighborhoods with a greater percentage of Black residents (adjusted odds ratio [a*OR*] = 0.96, 95% confidence interval [CI; 0.95, 0.97]), Hispanic or Latino residents (a*OR* = 0.97, CI [0.96, 0.98]), and vacant housing units (a*OR* = 0.75, CI [0.73, 0.77]) had a lower odds of having a tobacco-selling pharmacy. A similar pattern of results was observed for the presence of a tobacco shop. On the other hand, tract-level 150% FPL was associated with greater odds of a tract having a pharmacy (a*OR* = 1.03, CI [1.01, 1.04]) and tobacco shop (a*OR* = 1.18, CI [1.16, 1.20]).

**Table 4.** Analyses Testing Tract-Level Associations of Percentage Sociodemographics With the Presence (vs. Absence) of a Tobacco-Selling Pharmacy or Tobacco Shop, United States, 2018 (*N* = 71,495).

Sociodemographic	Presence of tobacco-selling pharmacy				Presence of tobacco shop			
	Unadjusted		Adjusted		Unadjusted		Adjusted	
	*OR*	95% CI	a*OR*	95% CI	*OR*	95% CI	a*OR*	95% CI
Non-Hispanic Black	0.97	[0.96, 0.98]	0.96	[0.95, 0.97]	0.93	[0.92, 0.94]	0.87	[0.86, 0.89]
Hispanic or Latino	0.98	[0.97, 0.99]	0.97	[0.96, 0.98]	1.03	[1.02, 1.04]	0.95	[0.93, 0.96]
Living below 150% FPL	0.96	[0.95, 0.97]	1.03	[1.01, 1.04]	1.06	[1.05, 1.07]	1.18	[1.16, 1.20]
Vacant housing units	0.82	[0.81, 0.84]	0.75	[0.73, 0.77]	0.88	[0.86, 0.90]	0.83	[0.81, 0.85]
Urbanicity								
Urban	—		Reference		—		Reference	
Large rural city/town	—		1.02	[0.96, 1.09]	—		0.98	[0.92, 1.05]
Small and isolated rural town	—		0.99	[0.92, 1.06]	—		0.65	[0.59, 0.70]

*Note.* Unadjusted logistic regression models tested the association between each sociodemographic variable (rows) and the outcome variables (column). Adjusted models control for tract-level urbanicity and other sociodemographics (% Black, Hispanic or Latino, living below 150% FPL, vacant housing) and include a state fixed effect. Tract-level sociodemographic variables were scaled to 10s (e.g., 10% is coded 1.0). CI = confidence interval; OR = odds ratio; FPL = federal poverty level.

## Discussion

In 2018, tobacco retailer density differed by neighborhood characteristics, but the extent and direction of these associations were sometimes sensitive to the density measure used. Neighborhoods with a greater percentage of residents living below 150% FPL were associated with higher tobacco retailer density in all models. In our results, however, the direction and significance of adjusted associations between retailer density and percentage of non-Hispanic Black residents, Hispanic or Latino residents, and vacant housing units were sensitive to the density measure operationalized.

Several U.S. studies have documented greater retailer density in neighborhoods with a higher percentage of Black residents (Hyland et al., 2003; Rodriguez et al., 2013). Our unadjusted results concur with these findings for all retailer density measures. However, in adjusted models, this association was negative for both total count of retailers and the number of retailers per 1,000 people. Although this finding may suggest that there is less tobacco availability in neighborhoods with a greater percentage of Black residents, adjusted results represent what would be observed in a counterfactual scenario where confounding sociodemographic variables in the model were held constant. Therefore, given that all unadjusted and some adjusted models indicated an inequity by percentage Black, greater retailer availability in these neighborhoods is still a concern in the real world. Similarly, a higher neighborhood percentage of Hispanic or Latino residents was associated with a greater total count of retailers, retailers per square mile, and retailers per 10 km of roadway in unadjusted models. In adjusted models, a higher neighborhood percentage of Hispanic or Latino residents was associated with fewer retailers per 1,000 people. The inverse adjusted associations between percentage Black and Hispanic or Latino residents and retailers per 1,000 people are consistent with a 2012 U.S. study limited to a sample of tracts within 97 counties (Lee et al., 2017). The tobacco industry has a long history of marketing tobacco products in the retail environment to Black and Latino and Hispanic individuals and communities (Iglesias-Rios & Parascandola, 2013; Kostygina et al., 2016; Yerger et al., 2007). Given associations of retailer availability and marketing with smoking behavior (Marsh et al., 2020; Robertson et al., 2015; Robertson et al., 2016; Valiente et al., 2020), greater tobacco retailer density in neighborhoods that have a higher percentage of these individuals is a public health concern.

We also found that for tracts with a 10–percentage point difference in vacant housing, the model-estimated density difference was 0.12 tobacco retailers per 1,000 people, almost 11% of the average density in the sample. This positive association of greater vacant housing and greater tobacco retailer density was consistent with two other studies (Fakunle et al., 2018; Lee et al., 2017). However, for two other measures of retailer density (total count, per km roadway), we documented inverse associations with vacant housing. Though this study is cross-sectional and we cannot make inferences about changes in retailer density or demographics over time, we posit one reason for these findings. Vacant housing may reflect a relatively low population count in a place that historically housed more people and may therefore be structurally equipped with enough roads to accommodate more people. In places with a high percentage of vacant dwellings, retailers may not have a desire to invest in neighborhoods where there may be little demand. Fewer retailers in neighborhoods with the same number of historical roads might produce a reduction in total count and roadway-based density measures over time. Yet a drop in retailers that also corresponds with a population decrease could actually increase population-based retailer density measures depending on the relative drop in each. Longitudinal research assessing changes over time is needed to further shed light onto these potential processes.

In this study, we use different measures of retailer density to help increase comparability of findings with past and future studies assessing inequities in tobacco retailer density. Taken together, our findings indicate that while there are neighborhood sociodemographic differences in the availability of tobacco retailers, the nature of these differences is dependent on the measure of retailer density used. This may be particularly critical for local and state jurisdictions that are currently using measures of retailer density in policies designed to reduce retailer inequities. For example, San Francisco, California, limits the number of tobacco retailers to 45 in each supervisorial district while Philadelphia, Pennsylvania, limits the number of tobacco retailers to one per 1,000 people per planning district (ChangeLab Solutions, 2019). Although statistical criteria can indicate which measure may best fit specific models, these analyses may not be generalizable to other data. More important, each measure may capture different aspects of the tobacco built and social environment. For example, per capita measures may reflect different levels of consumer demand for retailers, land area measures may also describe spatial proximity of consumers to retailers, and roadway measures may indicate the ease with which consumers can access retailers via existing infrastructure.

The four measures of retailer density used in this study are not exhaustive, and some studies have used other more computationally advanced measures, such as fixed and adaptive kernel density, which are too complex to be used in local policies. Additionally, proximity-based measures, such as the distance of tobacco retailers to one another, may be important for further understanding inequities in tobacco retailer availability. Which of these measures might most influence exposure to tobacco marketing, purchasing behavior, and tobacco use, however, requires greater research on how people interact within their activity spaces (Marsh et al., 2020; Valiente et al., 2020). Additionally, careful consideration should be taken when thinking about which measures might be most feasible to calculate or interpretable for communities and policymakers.

Given that policies regulating the sale of tobacco products in both pharmacies and tobacco shops have been implemented in several jurisdictions to reduce tobacco retailer density, we investigated whether neighborhood demographics were associated with the presence of tobacco-selling pharmacies or tobacco shops. We found that there was a lower odds of a tract having a tobacco-selling pharmacy in neighborhoods with a greater percentage of Black or Hispanic or Latino residents in adjusted models, suggesting that policies that restrict the sales of tobacco products in pharmacies may not decrease the number of tobacco retailers equitably across neighborhoods. These findings are consistent with other studies that have investigated the impact of a pharmacy ban on inequities in tobacco retailer density (Craigmile et al., 2020; Giovenco et al., 2019; Kulbicki & Leslie, 2015; Tucker-Seeley et al., 2016). While our study sample was limited to pharmacies that sell tobacco products, our findings are similar to those focused on pharmacy deserts, which find that pharmacies are less available in neighborhoods with a greater proportion of some racially and ethnically minoritized groups (Qato et al., 2014). Finally, the policy focus to date has been on restricting sales of tobacco products in pharmacies; however, one New Zealand modeling study considered the impact of only *permitting* sales of tobacco products in pharmacies (Petrovic-van der Deen et al., 2019). Our results indicate that this type of policy, if implemented in the United States, may result in less tobacco availability in neighborhoods with a higher percentage of Black or Hispanic or Latino residents.

Compared to White individuals who smoke, Black individuals who smoke are less likely to purchase cigarettes at tobacco shops (Groom et al., 2020), and our study found that the odds of a tract having at least one tobacco shop (vs. none) was lower in neighborhoods with a greater percentage of Black residents. Additionally, we found that the odds of a tract having a tobacco shop (vs. none) was higher in areas with a greater percentage of residents living below 150% FPL. Notably, the odds of having a tobacco shop was larger (a*OR* = 1.18, 95% CI [1.16, 1.20]) than the odds of having a tobacco-selling pharmacy (a*OR* = 1.03, CI [1.01, 1.04]) for tract-level 150% FPL, which may be especially concerning as tobacco shops primarily sell tobacco products while tobacco-selling pharmacies sell other basic goods and health-promoting items. Some jurisdictions have implemented policies that allow flavored tobacco products to be sold only in adult-only tobacco shops. If flavored tobacco products, or possibly any tobacco products, are permitted to be sold only in tobacco shops, people living in neighborhoods with a greater percentage of Black residents may have less tobacco product availability, yet those in neighborhoods with a greater percentage of residents living below 150% FPL may have more. Policymakers may want to consider the availability of certain tobacco retailer types in their communities when implementing retailer reduction and/or product availability regulations, as these regulations may have a differential impact on tobacco product availability for some neighborhoods.

Several considerations should be made when interpreting the results of this study. First, we created a probable list of tobacco retailers, and it is possible that our list contained retailers that do not sell tobacco, or there could be tobacco retailers missing. We have no reason to believe that this potential error is systematic. Second, as our analytic sample represents a near census of all tracts in the United States, we have high power to detect small associations, and caution should be taken when interpreting small effect sizes. On the other hand, because this is a near census of tracts, associations observed may be more likely to represent the “true” population parameter. Of importance is that we conceptualized the neighborhood as a census tract, but other neighborhood scales (e.g., block groups) may be appropriate. Additionally, there may be other important neighborhood sociodemographic characteristics that may contribute to the associations observed (e.g., commercial land use) that deserve future investigation. Finally, this study is cross-sectional, and temporality cannot be established. Regardless of temporality, tobacco products are not a health-promoting neighborhood commodity, and their availability and marketing could influence smoking behaviors.

In this national study, we use several common measures of tobacco retailer density to investigate associations with tract-level sociodemographic characteristics. While we document inequities in retailer density by area race, ethnicity, poverty, and vacant housing, these relationships were not consistent across all measures. Researchers and policymakers should consider how various measures of tobacco retailer density may capture different aspects of the tobacco retailer environment in their communities. Furthermore, attention to whether the availability of certain tobacco retailer types differs across and by neighborhood characteristics may be important when considering the varying impact of some tobacco retailer reduction policies. Overall, identifying the relationships between neighborhood sociodemographic characteristics and tobacco retailer availability may help communities better track place-based tobacco retailer inequities and design impactful pro-equity retailer-focused strategies.

## Supplemental Material

sj-docx-1-heb-10.1177_10901981211008390 – Supplemental material for Neighborhood Inequities in Tobacco Retailer Density and the Presence of Tobacco-Selling Pharmacies and Tobacco Shops Click here for additional data file. Supplemental material, sj-docx-1-heb-10.1177_10901981211008390 for Neighborhood Inequities in Tobacco Retailer Density and the Presence of Tobacco-Selling Pharmacies and Tobacco Shops by Amanda Y. Kong, Paul L. Delamater, Nisha C. Gottfredson, Kurt M. Ribisl, Chris D. Baggett and Shelley D. Golden in Health Education & Behavior

## References

[bibr1-10901981211008390] Aiyer S. M. Zimmerman M. A. Morrel-Samuels S. Reischl T. M. (2015). From broken windows to busy streets: A community empowerment perspective. Health Education & Behavior, 42(2), 137–147. 10.1177/1090198114558590 25512073

[bibr2-10901981211008390] Browning C. R. Cagney K. A. (2003). Moving beyond poverty: Neighborhood structure, social processes, and health. Journal of Health and Social Behavior, 44(4), 552–571. 10.2307/1519799 15038149

[bibr3-10901981211008390] Chaiton M. O. Mecredy G. C. Cohen J. E. Tilson M. L. (2013). Tobacco retail outlets and vulnerable populations in Ontario, Canada. International Journal of Environmental Research and Public Health, 10(12), 7299–7309. 10.3390/ijerph10127299 24351748 PMC3881168

[bibr4-10901981211008390] ChangeLab Solutions. (2019). Tobacco retailer density: Place-based strategies to advance health and equity. https://www.changelabsolutions.org/sites/default/files/CLS-BG214-Tobacco_Retail_Density-Factsheet_FINAL_20190131.pdf

[bibr5-10901981211008390] CounterTobacco.org. (2019). Tobacco free pharmacies. https://countertobacco.org/policy/tobacco-free-pharmacies/

[bibr6-10901981211008390] CounterTobacco.org. (2020). Restricting product availability. https://countertobacco.org/policy/restricting-product-availability/

[bibr7-10901981211008390] Craigmile P. F. Onnen N. Schwartz E. Glasser A. Roberts M. E. (2020). Evaluating how licensing-law strategies will impact disparities in tobacco retailer density: A simulation in Ohio. Tobacco Control. Advance online publication. 10.1136/tobaccocontrol-2020-055622 PMC7897331 32826386

[bibr8-10901981211008390] Dai H. Hao J. Catley D. (2017). Vape shop density and socio-demographic disparities: A US Census tract analysis. Nicotine & Tobacco Research, 19(11), 1338–1344. 10.1093/ntr/ntx063 28371830

[bibr9-10901981211008390] Fakunle D. O. Thorpe R. J. Jr. Furr-Holden C. D. M. Curriero F. C. Leaf P. J. (2018). Does tobacco outlet inequality extend to high-White Mid-Atlantic jurisdictions? A study of socioeconomic status and density. Journal of Racial and Ethnic Health Disparities, 6, 409–418. 10.1007/s40615-018-00538-9 30446987 PMC6424620

[bibr10-10901981211008390] Finan L. J. Lipperman-Kreda S. Abadi M. Grube J. W. Kaner E. Balassone A. Gaidus A. (2019). Tobacco outlet density and adolescents’ cigarette smoking: A meta-analysis. Tobacco Control, 28(1), 27–33. 10.1136/tobaccocontrol-2017-054065 29519934 PMC6129215

[bibr11-10901981211008390] Giovenco D. P. Spillane T. E. Mauro C. M. Hernandez D. (2019). Evaluating the impact and equity of a tobacco-free pharmacy law on retailer density in New York City neighbourhoods. Tobacco Control, 28(5), 548–554. 10.1136/tobaccocontrol-2018-054463 30135112 PMC6597322

[bibr12-10901981211008390] Groom A. L. Cruz-Cano R. Mead E. L. Giachello A. L. Hart J. L. Walker K. L. Oncken C. Robertson R. M. (2020). Tobacco point-of-sale influence on U.S. adult smokers. Journal of Health Care for the Poor and Underserved, 31(1), 249–264. 10.1353/hpu.2020.0021 32037330 PMC12991829

[bibr13-10901981211008390] Hall J. Cho H. D. Maldonado-Molina M. George T. J. Shenkman E. A. Salloum R. G. (2019). Rural-urban disparities in tobacco retail access in the southeastern United States: CVS vs. the dollar stores. Preventive Medicine Reports, 15, 100935. 10.1016/j.pmedr.2019.100935 PMC6637220 31360628

[bibr14-10901981211008390] Hyland A. Travers M. J. Cummings M. Bauer J. Alford T. Wieczorek W. F. (2003). Tobacco outlet density and demographics in Erie County, New York. American Journal of Public Health, 93(7), 1075–1076. 10.2105/AJPH.93.7.1075 12835184 PMC1447908

[bibr15-10901981211008390] Iglesias-Rios L. Parascandola M. (2013). A historical review of R. J. Reynolds’ strategies for marketing tobacco to Hispanics in the United States. American Journal of Public Health, 103(5), e15–e27. 10.2105/AJPH.2013.301256 PMC3698830 23488493

[bibr16-10901981211008390] Inwood J. F. Yarbrough R. A. (2010). Racialized places, racialized bodies: The impact of racialization on individual and place identities. GeoJournal, 75(3), 299–301. 10.1007/s10708-009-9308-3

[bibr17-10901981211008390] Kawachi I. Berkman L. (2000). Social cohesion, social capital, and health. In Kawachi I. Berkman L. (Eds.), Social epidemiology (pp. 174–190). Oxford University Press.

[bibr18-10901981211008390] Kite J. Rissel C. Greenaway M. Willliams K. (2014). Tobacco outlet density and social disadvantage in New South Wales, Australia. Tobacco Control, 23(2), 181–182. 10.1136/tobaccocontrol-2012-050648 23242978 PMC3932954

[bibr19-10901981211008390] Kong A. Y. Baggett C. D. Gottfredson N. C. Ribisl K. M. Delamater P. L. Golden S. D. (2021). Associations of tobacco retailer availability with chronic obstructive pulmonary disease related hospital outcomes, United States, 2014. Health & Place, 67, 102464. 10.1016/j.healthplace.2020.102464 PMC7854476 33276261

[bibr20-10901981211008390] Kong A. Y. Myers A. E. Isgett L. F. Ribisl K. M. (2020). Neighborhood racial, ethnic, and income disparities in accessibility to multiple tobacco retailers: Mecklenburg County, North Carolina, 2015. Preventive Medicine Reports, 17, 101031. 10.1016/j.pmedr.2019.101031 PMC6993011 32021758

[bibr21-10901981211008390] Kostygina G. Glantz S. A. Ling P. M. (2016). Tobacco industry use of flavours to recruit new users of little cigars and cigarillos. Tobacco Control, 25(1), 66–74. 10.1136/tobaccocontrol-2014-051830 25354674 PMC4414663

[bibr22-10901981211008390] Krieger N. Waterman P. D. Gryparis A. Coull B. A. (2015). Black carbon exposure, socioeconomic and racial/ethnic spatial polarization, and the Index of Concentration at the Extremes (ICE). Health & Place, 34(July), 215–228. 10.1016/j.healthplace.2015.05.008 26093080 PMC4681506

[bibr23-10901981211008390] Kulbicki K. M. Leslie T. F. (2015). The effect on spatial accessibility from removing tobacco from pharmacies in the Washington, DC area. Public Health, 129(9), 1285–1287. 10.1016/j.puhe.2015.07.006 26318616

[bibr24-10901981211008390] Lee J. G. L. Schleicher N. C. Leas E. C. Henriksen L. (2018). US Food and Drug Administration inspection of tobacco sales to minors at top pharmacies, 2012-2017. JAMA Pediatrics, 172(11), 1089–1090. 10.1001/jamapediatrics.2018.2150 30193340 PMC6248162

[bibr25-10901981211008390] Lee J. G. L. Sun D. L. Schleicher N. M. Ribisl K. M. Luke D. A. Henriksen L. (2017). Inequalities in tobacco outlet density by race, ethnicity and socioeconomic status, 2012, USA: Results from the ASPiRE Study. Journal of Epidemiology & Community Health, 71(5), 487–492. 10.1136/jech-2016-208475 28249990 PMC5458784

[bibr26-10901981211008390] Lipsitz G. (2007). The racialization of space and the spatialization of race: Theorizing the hidden architecture of landscape. Landscape Journal, 26(1), 10–23. 10.3368/lj.26.1.10

[bibr27-10901981211008390] Marsh L. Vaneckova P. Robertson L. Johnson T. O. Doscher C. Raskind I. G. Schleicher N. C. Henriksen L. (2020). Association between density and proximity of tobacco retail outlets with smoking: A systematic review of youth studies. Health & Place, 67, 102275. 10.1016/j.healthplace.2019.102275 PMC8171582 33526204

[bibr28-10901981211008390] Mayers R. S. Wiggins L. L. Fulghum F. H. Peterson N. A. (2012). Tobacco outlet density and demographics: A geographically weighted regression analysis. Prevention Science, 13(5), 462–471. 10.1007/s11121-011-0273-y 22538505

[bibr29-10901981211008390] Neely B. Samura M. (2011). Social geographies of race: Connecting race and space. Ethnic and Racial Studies, 34(11), 1933–1952. 10.1080/01419870.2011.559262

[bibr30-10901981211008390] Peterson N. A. Lowe J. B. Reid R. J. (2005). Tobacco outlet density, cigarette smoking prevalence, and demographics at the county level of analysis. Substance Use & Misuse, 40(11), 1627–1635. 10.1080/10826080500222685 16253931

[bibr31-10901981211008390] Petrovic-van der Deen F. S. Blakely T. Kvizhinadze G. Cleghorn C. L. Cobiac L. J. Wilson N. (2019). Restricting tobacco sales to only pharmacies combined with cessation advice: A modelling study of the future smoking prevalence, health and cost impacts. Tobacco Control, 28(6), 643–650. 10.1136/tobaccocontrol-2018-054600 30413563

[bibr32-10901981211008390] Pratto F. Sidanius J. Levin S. (2006). Social dominance theory and the dynamics of intergroup relations: Taking stock and looking forward. European Review of Social Psychology, 17(1), 271–320. 10.1080/10463280601055772

[bibr33-10901981211008390] Qato D. M. Daviglus M. L. Wilder J. Lee T. Qato D. Lambert B. (2014). ‘Pharmacy deserts’ are prevalent in Chicago’s predominantly minority communities, raising medication access concerns. Health Affairs, 33(11), 1958–1965. 10.1377/hlthaff.2013.1397 25367990

[bibr34-10901981211008390] Ribisl K. M. D’Angelo H. Feld A. L. Schleicher N. C. Golden S. D. Luke D. A. Henriksen L. (2017). Disparities in tobacco marketing and product availability at the point of sale: Results of a national study. Preventive Medicine, 105, 381–388. 10.1016/j.ypmed.2017.04.010 28392252 PMC5630502

[bibr35-10901981211008390] Robertson L. Cameron C. McGee R. Marsh L. Hoek J. (2016). Point-of-sale tobacco promotion and youth smoking: A meta-analysis. Tobacco Control, 25(e2), e83. 10.1136/tobaccocontrol-2015-052586 26728139

[bibr36-10901981211008390] Robertson L. McGee R. Marsh L. Hoek J. (2015). A systematic review on the impact of point-of-sale tobacco promotion on smoking. Nicotine & Tobacco Research, 17(1), 2–17. 10.1093/ntr/ntu168 25173775 PMC4832971

[bibr37-10901981211008390] Rodriguez D. Carlos H. A. Adachi-Mejia A. M. Berke E. M. Sargent J. D. (2013). Predictors of tobacco outlet density nationwide: A geographic analysis. Tobacco Control, 22(5), 349–355. 10.1136/tobaccocontrol-2011-050120 22491038 PMC3431432

[bibr38-10901981211008390] Rodriguez D. Carlos H. A. Adachi-Mejia A. M. Berke E. M. Sargent J. D. (2014). Retail tobacco exposure: Using geographic analysis to identify areas with excessively high retail density. Nicotine & Tobacco Research, 16(2), 155–165. 10.1093/ntr/ntt126 23999651 PMC3880231

[bibr39-10901981211008390] Schneider J. E. Reid R. J. Peterson N. A. Lowe J. B. Hughey J. (2005). Tobacco outlet density and demographics at the tract level of analysis in Iowa: Implications for environmentally based prevention initiatives. Prevention Science, 6(4), 319–325. 10.1007/s11121-005-0016-z 16163568

[bibr40-10901981211008390] Shortt N. K. Tisch C. Pearce J. Mitchell R. Richardson E. A. Hill S. Collin J. (2015). A cross-sectional analysis of the relationship between tobacco and alcohol outlet density and neighbourhood deprivation. BMC Public Health, 15(1), Article 1014. 10.1186/s12889-015-2321-1 PMC4595054 26437967

[bibr41-10901981211008390] Siahpush M. Jones P. R. Singh G. K. Timsina L. R. Martin J. (2010). The association of tobacco marketing with median income and racial/ethnic characteristics of neighbourhoods in Omaha, Nebraska. Tobacco Control, 19(3), 256–258. 10.1136/tc.2009.032185 20395407

[bibr42-10901981211008390] Stockdale S. E. Wells K. B. Tang L. Belin T. R. Zhang L. Sherbourne C. D. (2007). The importance of social context: Neighborhood stressors, stress-buffering mechanisms, and alcohol, drug, and mental health disorders. Social Science & Medicine, 65(9), 1867–1881. 10.1016/j.socscimed.2007.05.045 17614176 PMC2151971

[bibr43-10901981211008390] Tucker-Seeley R. D. Bezold C. P. James P. Miller M. Wallington S. F. (2016). Retail pharmacy policy to end the sale of tobacco products: What is the impact on disparity in neighborhood density of tobacco outlets? Cancer Epidemiology, Biomarkers & Prevention, 25(9), 1305–1310. 10.1158/1055-9965.EPI-15-1234 PMC5010482 27302724

[bibr44-10901981211008390] U.S. Department of Agriculture. (2016). 2010 Rural-Urban Commuting Area (RUCA) Codes documentation. https://www.ers.usda.gov/data-products/rural-urban-commuting-area-codes/documentation/

[bibr45-10901981211008390] Valiente R. Escobar F. Urtasun M. Franco M. Shortt N. K. Sureda X. (2020). Tobacco retail environment and smoking: A systematic review of geographic exposure measures and implications for future studies. Nicotine & Tobacco Research. Advance online publication. 10.1093/ntr/ntaa223 33155040

[bibr46-10901981211008390] Yerger V. B. Przewoznik J. Malone R. E. (2007). Racialized geography, corporate activity, and health disparities: Tobacco industry targeting of inner cities. Journal of Health Care for the Poor and Underserved, 18(4 Suppl.), 10–38. 10.1353/hpu.2007.0120 18065850

