# Supplementary material for: Neighborhood Inequities in Tobacco Retailer Density and the Presence of Tobacco-Selling Pharmacies and Tobacco Shops
Source: Health Educ Behav. 2021 Apr 19;49(3):478–87. doi: 10.1177/10901981211008390 (PMC8523582; doi:10.1177/10901981211008390)
Supplement: sj-docx-1-heb-10.1177_10901981211008390 – Supplemental material for Neighborhood Inequities in Tobacco Retailer Density and the Presence of Tobacco-Selling Pharmacies and Tobacco Shops [file sj-docx-1-heb-10.1177_10901981211008390.docx]

**Appendix A:** Estimated Simple Slopes of Census Tract-Level Associations of Percent Sociodemographics with Measures of Tobacco Retailer Availability by Urbanicity, United States, 2018 (N=71,495)

|  | **Total count**  **of retailers** | | **Retailers per**  **1000 people** | | **Retailers per**  **square mile** | | **Retailers per 10 km of roadway** | |
| --- | --- | --- | --- | --- | --- | --- | --- | --- |
|  | **B (SE)** |  | **B (SE)** |  | **B (SE)** |  | **B (SE)** |  |
| Small & Isolated Rural Town (n=6059) |  |  |  |  |  |  |  |  |
| Non-Hispanic Black | **0.19 (0.05)** | U | **0.07 (0.01**) | U | 0.05 (0.14) |  | 0.03 (0.02) |  |
| Hispanic or Latino | **0.34 (0.05)** | LR, U | **0.05 (0.01)** | LR, U | 0.04 (0.15) | U | 0.02 (0.03) | U |
| Living below 150% FPL | **0.57 (0.06)** | U | **0.19 (0.01**) | U | 0.14 (0.15) | LR, U | **0.07 (0.03)** | LR, U |
| Vacant housing units | **-0.47 (0.04)** | U | **0.06 (0.01)** | LR, U | -0.09 (0.10) |  | **-0.06 (0.02)** |  |
| Large Rural City/Town (n=6204) |  |  |  |  |  |  |  |  |
| Non-Hispanic Black | 0.08 (0.04) | U | **0.06 (0.01)** | U | 0.13 (0.12) |  | **0.05 (0.02)** |  |
| Hispanic or Latino | **0.18 (0.04)** | SR, U | 0.02 (0.01) | SR, U | 0.16 (0.11) | U | **0.05 (0.02**) | U |
| Living below 150% FPL | **0.47 (0.05)** | U | **0.18 (0.01)** | U | **0.66 (0.13)** | SR, U | **0.16 (0.02)** | SR, U |
| Vacant housing units | -**0.40 (0.05)** | U | **0.10 (0.01)** | SR, U | -0.06 (0.13) |  | **-0.05 (0.02)** |  |
| Urban (n=59,232) |  |  |  |  |  |  |  |  |
| Non-Hispanic Black | **-0.15 (0.01)** | SR, LR | **-0.03 (0.00)** | SR, LR | 0.14 (0.03) |  | **0.03 (0.00)** |  |
| Hispanic or Latino | 0.02 (0.01) | SR, LR | **-0.02 (0.00)** | SR, LR | **0.96 (0.03)** | SR, LR | **0.17 (0.01)** | SR, LR |
| Living below 150% FPL | **0.37 (0.01)** | SR, LR | **0.14 (0.00)** | SR, LR | **1.37 (0.04)** | SR, LR | **0.27 (0.01)** | SR, LR |
| Vacant housing units | **-0.06 (0.02)** | SR, LR | **0.15 (0.01)** | SR, LR | 0.01 (0.06) |  | **-0.05 (0.01)** |  |

Note: In a post-hoc multivariable analysis, we included interaction terms between each sociodemographic variable and the three-level urbanicity variable. We report simple slope estimates (B) for each urbanicity category (boldface indicates statistical significance, p<0.05). We further tested whether simple slope estimates significantly differed (p<0.05) between each level of urbanicity (SR=significant difference from Small and Isolated Rural Town; LR= significant difference from Large Rural City/Town; U= significant difference from Urban area). All models control for other sociodemographics (% Black, Hispanic or Latino, living below 150% FPL, vacant housing) and include a state fixed effect. Tract-level sociodemographic variables were scaled to 10s (e.g., 10% is coded 1.0) so that estimates may be interpreted as the expected difference in tobacco retailer availability for a census tract that has a 10-percentage point greater value in the sociodemographic variable.

FPL = Federal Poverty Level
